# Successful recanalization of acute extensive portal vein thrombosis by aspiration thrombectomy and thrombolysis via an operatively placed mesenteric catheter: a case report

**DOI:** 10.1259/bjrcr.20180022

**Published:** 2018-05-21

**Authors:** Norifumi Kennoki, Toru Saguchi, Toru Sano, Tomohisa Moriya, Natsuhiko Shirota, Jun Otaka, Kunihito Suzuki, Koichi Tomita, Naokazu Chiba, Shigeyuki Kawachi, Kiyoshi Koizumi, Koichi Tokuuye

**Affiliations:** 1 Department of Radiology, Tokyo Medical University Hospital, Tokyo, Japan; 2 Department of Digestive and Transplantation Surgery, Tokyo Medical University Hachioji Medical Center, Tokyo, Japan; 3 Department of Radiology, Tokyo Medical University Hachioji Medical Center, Tokyo, Japan

## Abstract

Portal vein thrombosis (PVT) after hepatobiliary surgery is rare but can cause lethal and severe complications. If early diagnosis and recanalization can be achieved, the PVT is expected to be eliminated. A 70-year-old male was diagnosed as having hepatocellular carcinoma occupying the right lobe of the liver. As oligometastatic lung tumors were simultaneously detected on contrast-enhanced CT (CECT), hepatectomy was not indicated. However, the primary tumor was very large, and as large tumor size can be associated with an unfavorable prognosis, and owing to the strong desire of the patient, he underwent right lobe hepatectomy. Jaundice appeared on post-operative Day (POD) 2 and CECT displayed slight intraheptatic bile duct dilation. However, a PVT did not exist at this time. Percutaneous transhepatic biliary drainage was performed and Doppler echo displayed intrahepatic and extrahepatic PVT on post-operative Day 5. Emergent thrombectomy was performed using a Vasplyser Plus^TM^ thrombus aspiration catheter (Johnson & Johnson K.K. Medical Company, Tokyo, Japan) via the ileocolic vein under laparotomy. The mesenteric catheter was placed at the distal point of the residual PVT. Thrombolysis and anticoagulant therapy were performed using heparin and urokinase. In the CECT performed 16 days after the additional operation, the PVT had disappeared and the portal vein was completely recanalized. The mesenteric catheter was removed on the same day and oral anticoagulant therapy was continued. At the time of writing, 14 months have passed with no recurrence of PVT. Early diagnosis of PVT enables treatment with emergent thrombectomy, thrombolysis, and anticoagulant therapy. These treatments result in the improvement of portal vein flow and the complete disappearance of PVT.

## Background

The standard therapy for portal vein thrombosis (PVT) is systemic anticoagulant therapy. However, most thrombi (both intra- and extrahepatic) are often not sufficiently dissolved by only systemic anticoagulant therapy. Recently, in addition to systemic anticoagulation therapy, interventional treatments, such as aspiration thrombectomy and continuous thrombolytic therapy using an indwelling mesenteric catheter have been reported to be useful.^1–4^ Here, we report a case in which acute PVT was found incidentally after hepatectomy, and successful recanalization was achieved by rapid interventional treatment.

## Case report

**Figure 1.**
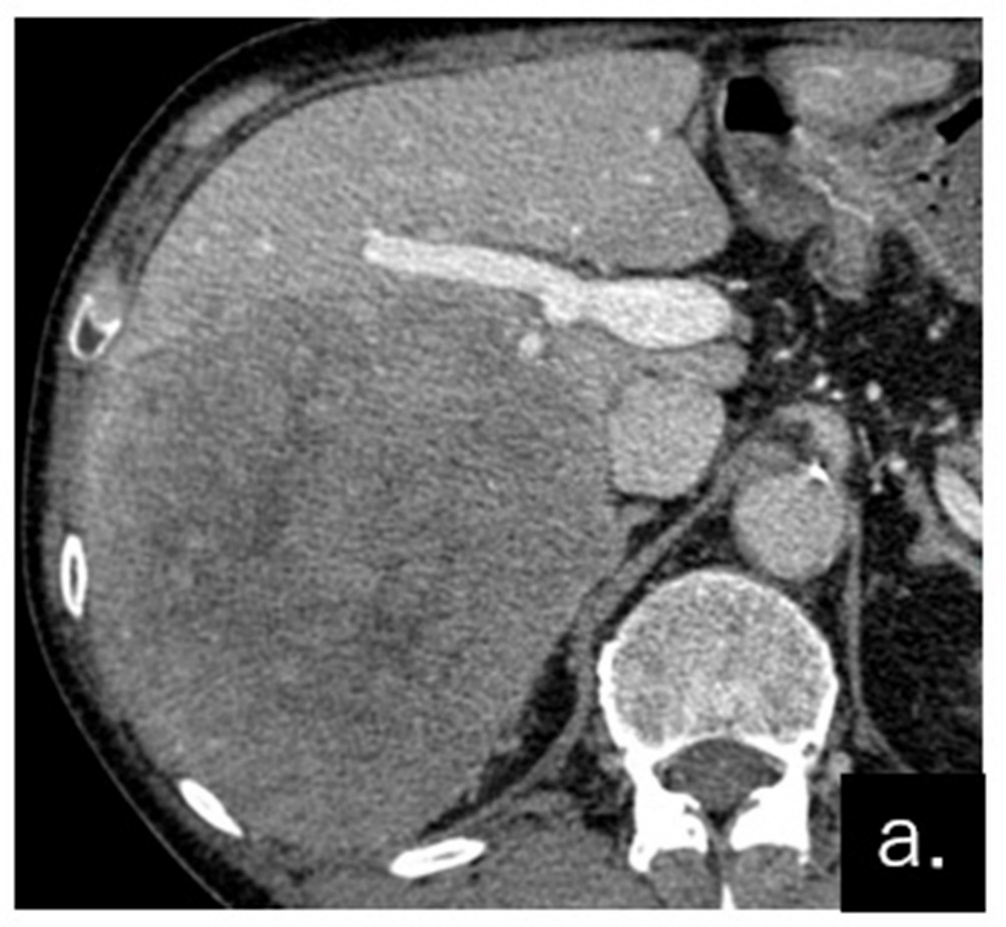
Contrast-enhanced CT (CECT) of the liver before hepatectomy. The primary tumor (hepatocellular carcinoma) occupied the right lobe of the liver.

Our study was exempted from ethical approval by our institutional review board (Tokyo Medical University Hachioji Medical Center, Tokyo). Written informed consent for the case to be published (including images, case history, and data) was obtained from the patient for publication of this case report, including accompanying images. A 70-year-old male visited the gastrointestinal department of our hospital with chief complaints of anorexia and weight loss. The patient had no obvious history of chronic liver disease. Contrast-enhanced CT (CECT) displayed a large tumor of 130 × 120 mm in the liver, with early staining and washout (Figure 1). Laboratory data showed that levels of alpha-fetoprotein and protein induced by vitamin K absence/antagonist–II were increased to more than 20,000 ng ml^−1^ and 80,000 mAU ml^–1^, respectively. The patient was thus clinically diagnosed as having hepatocellular carcinoma (HCC). An enlarged hepatic hilar lymph node and oligometastatic lung tumors were also observed, and therefore, hepatectomy was not indicated. However, as a large primary HCC can be associated with an unfavorable prognosis, and owing to the patient’s strong wish, a hepatectomy was performed. The patient was pathologically diagnosed as moderately differentiated HCC T4NXM1 Stage IVb. The noncancerous liver tissue was normal (no fibrosis was observed).

**Figure 2.**
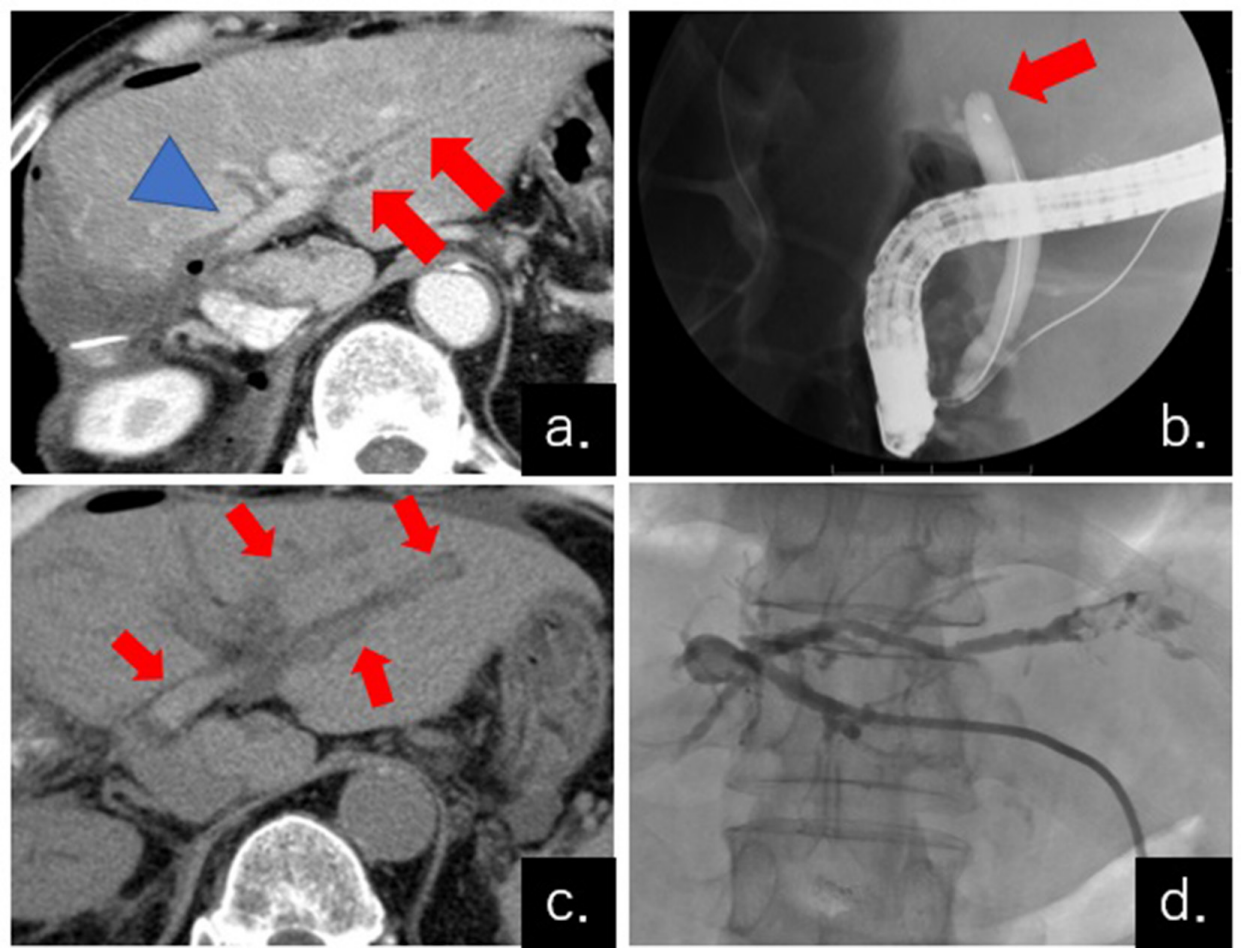
CT and fluorography of the liver on POD 2 to 5. (a) Slight dilation of the intrahepatic bile duct was confirmed on POD 2 (arrows). No PVT was displayed at this time (arrowhead). (b) Endoscopic retrograde cholangiopancreatography was performed on POD 3. However, the guidewire could not be cannulated into the upper side of the common bile duct (arrow), which indicated an obstruction. (c) PVT was detected incidentally during the PTBD procedure on POD 5. The intrahepatic portal vein looked highly absorbed in the plain CT (arrows), which indicated a fresh thrombus. (d) Although PTBD was performed, the guidewire could not be cannulated. POD, post-operative day; PTBD, percutaneous transhepatic biliary drainage; PVT, portal vein thrombosis.

Jaundice appeared on post-operative Day 2 (POD 2), and intrahepatic bile duct dilation was confirmed by CECT. At that time, however, the PVT was not detected (Figure 2a). Although endoscopic retrograde cholangiopancreatography was performed, the upper bile duct was occluded and the guidewire could not be inserted (Figure 2b). When an abdominal Doppler echo was performed to puncture the dilated bile duct during percutaneous transhepatic biliary drainage (PTBD) on POD 5, no intrahepatic portal vein was detected. Plain CT was immediately performed and numerous portal vein thrombi were confirmed (Figure 2c). The PVT displayed high attenuation on plain CT, and was suggested to be an acute PVT. PTBD was performed promptly (Figure 2d), and reoperation combined with intervention was performed on the same day.

### Aspiration thrombectomy and placement of the mesenteric catheter

**Figure 3.**
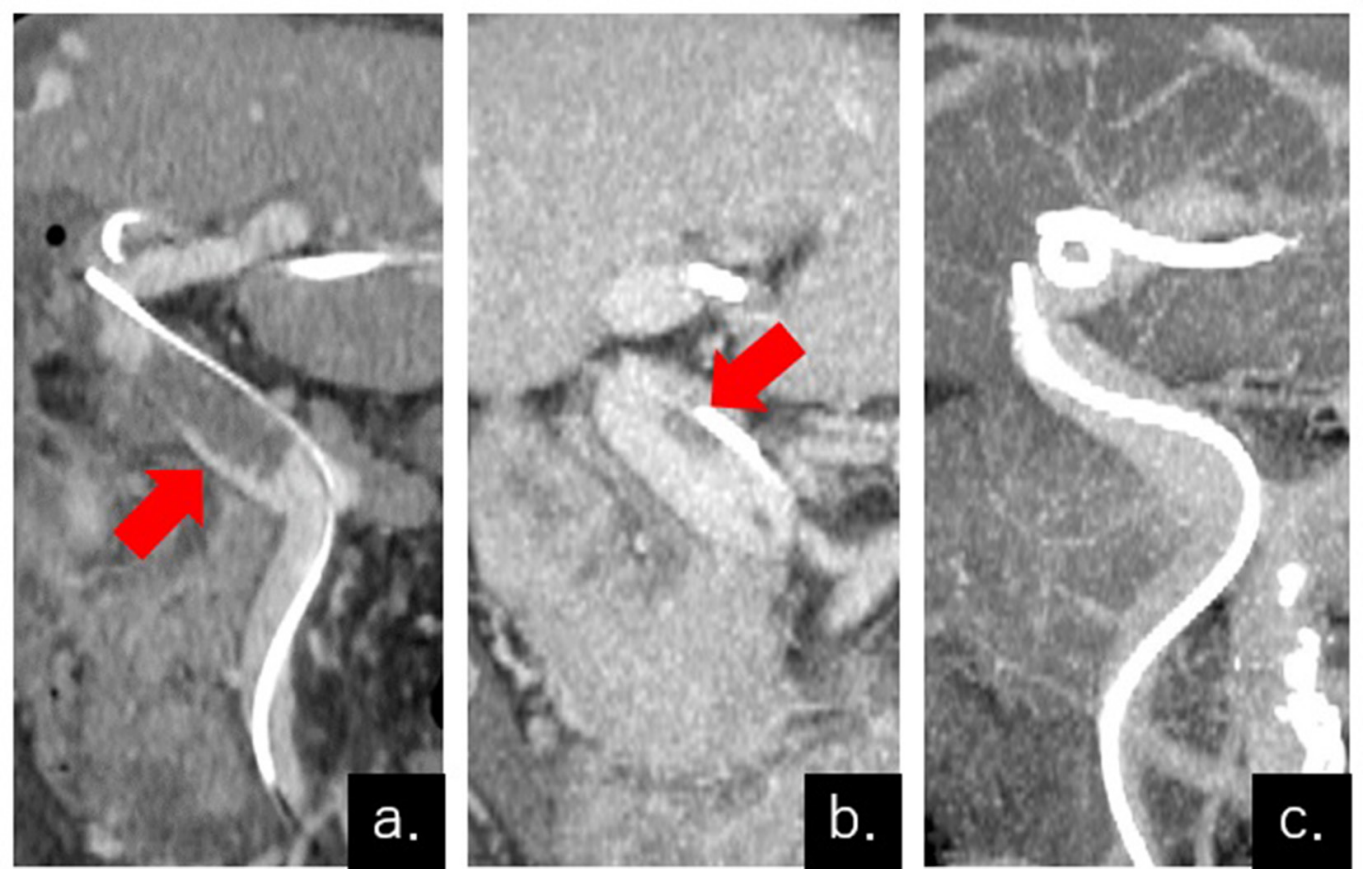
Comparison of the portal vein observed by CECT before reoperation and by fluorography during reoperation. (a) CECT image of the “defective part” shown in the main portal vein trunk indicating the thrombus (arrows). (b) Digital subtraction angiography image after insertion of a Destination™ guiding catheter. The “defective area” was in the same place as in the CECT image in (a) (arrows). (c) Thrombectomy and aspiration were performed using a Vasplyser Plus™ thrombus aspiration catheter and the portal vein was recanalized (arrows). CECT, contrast-enhanced CT.

**Figure 4.**
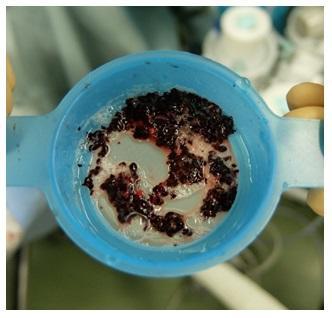
Gross appearance of the freshly aspirated thrombus. The aspirated thrombus was filtered through a mesh.

**Figure 5.**
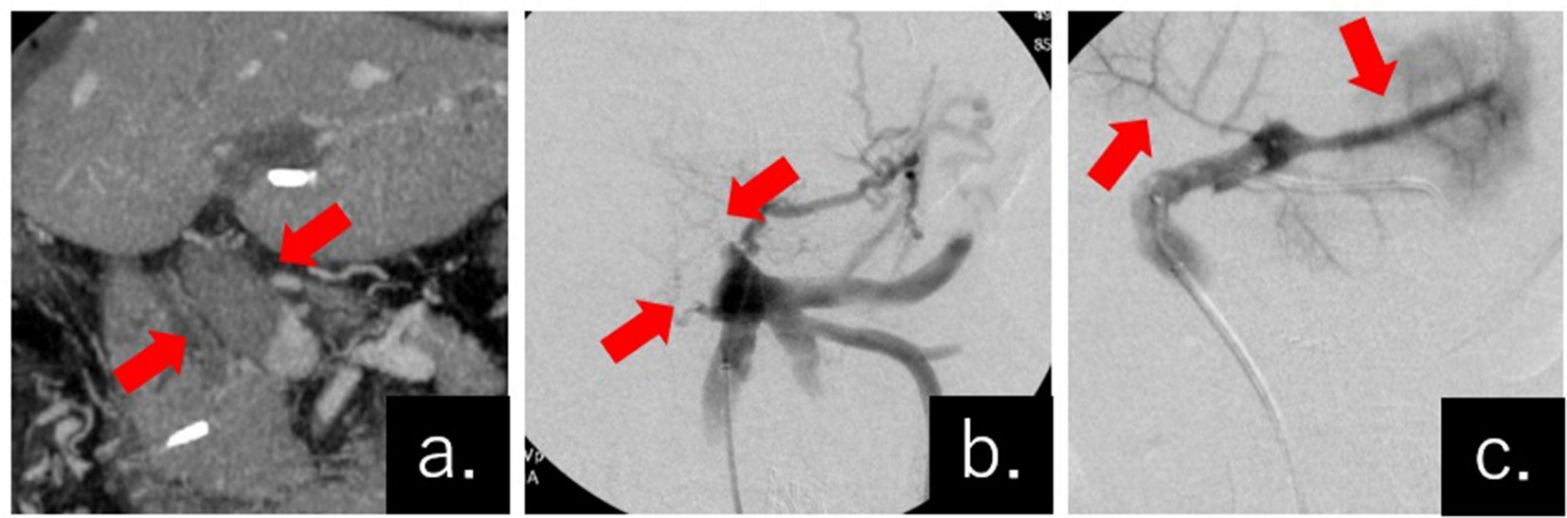
CECT of the liver after reoperation. The mesenteric catheter was placed on the distal side of the residual thrombus in the main trunk of the portal vein. CECT was displayed at 2 days (a), 9 days (b), and 16 days (c) after reoperation. Gradual thrombolysis was observed in the main trunk of the portal vein (arrows in a, b), and the residual thrombus was completely resolved 16 days after the reoperation (c). CECT, contrast-enhanced CT.

A 6-Fr Destination™ guiding catheter (TERUMO, Tokyo, Japan) and Vasplyser Plus™ thrombus aspiration catheter (Johnson & Johnson K.K. Medical Company, Tokyo, Japan) were inserted through the ileocolic vein under laparotomy. Thrombus crushing, aspiration, and removal were performed until blood flow in the peripheral portal vein was improved (Figure 3a,b,Figure 4). In the final imaging, improvement of outflow in the portal vein was confirmed (Figure 3c), and a part of the thrombus remained in the trunk of the portal vein. The tip of the mesenteric catheter was placed at the distal side of the residual thrombus (Figure 5a).

### Local thrombolysis, local and systemic anticoagulation therapy, and follow-up

As a thrombolytic therapy, 40,000 units day^−1^ of heparin and 300,000 units day^−1^ of urokinase were administered from the mesenteric catheter. As an anticoagulant therapy, 100,000 units day^–1^ of heparin was administered intravenously (i.v.). CECT 16 days after the additional operation showed that the PVT had completely disappeared (Figure 5c), and the mesenteric catheter was removed on the same day. i.v. heparin administration was also stopped and switched to 300 mg day^−1^ edoxaban orally. At the time of writing, the patient has been alive for 1 year and 2 months after the additional operation, and no further thrombosis nor portal hypertension have occurred.

## Discussion

Acute PVT after hepatectomy is found in 2–9% of patients, and hence it is relatively rare, but it is known to cause fatal and potential complications.^5, 6^ In general, the mechanism of thrombosis has been classically proposed as Virchow’s triad, *i.e.* venous stasis, hypercoagulable states, and endothelial injuries.^7^ The four major causes of acute PVT were reported to be right lobectomy, caudate lobectomy, splenectomy, and postoperative bile leakage.^5^ Right lobectomy and postoperative problems in the biliary system occurred in the present case. The portal vein after left lobectomy remains almost straight, whereas after right lobectomy, the portal vein tends to bend and kink, resulting in portal vein stasis, which can cause thrombus formation^8^ . Moreover, both long-time use of the Pringle maneuver and bile leakage are considered to be risk factors of endothelial injuries that form a thrombus. However, in the present case, an obvious kink in the portal vein was not displayed on follow-up CT, and the Pringle maneuver was used for a short time (26 min), and all surgical procedures were performed cautiously and quickly, with no major problems. Therefore, the only potential complication in the present case was problems in the biliary system. Biliary duct obstruction and inflammation were thought to have spread to the portal vein, similarly to that observed in cases of bile leakage, resulting in vascular endothelial injury, which subsequently contributed to thrombus formation.

Thrombi in the mesenteric system often extend to the superior mesenteric vein (SMV), and may cause various symptoms, such as abdominal pain, vomiting, and intestinal obstruction, which then develop into bowel ischemia or necrosis, sepsis, and death.^1, 9^ According to a previous report, catheter-directed thrombolytic therapy for eight acute SMV thrombosis patients were 100% successful and no patients died; however, four patients (50%) subsequently required intestinal resection.^2^ On the other hand, in cases in which the thrombus does not extend to the SMV, such as in the present case, no symptoms occur in many cases and discovery is delayed. When PVT is not appropriately treated in the acute phase, a thrombus can completely occlude the portal vein and cavernomas are formed and may result in fatal portal hypertension.^10^ Therefore, it is considered preferable to find and treat PVT as early as possible, irrespective of its state of progression to SMV. Furthermore, in general, because PVTs that have not yet progressed to SMV are asymptomatic, they are thought to be more difficult to detect and treat in the early stages than PVTs that have progressed to SMV.

Systemic anticoagulation therapy is recommended as a standard treatment for PVT.^11^ Condat et al reported that 31 acute PVT patients underwent systemic anticoagulation therapy, and recanalization was observed in 25 patients (80.6%). This included complete, partial, and no recanalization in 10 (32.3%), 15 (48.3%), and 2 (6.5%) patients, respectively, and the other 2 patients did not undergo follow-up CT. Complete recanalization was achieved more frequently in patients with limited portal vein or SMV thrombosis than in patients with extensive portal system thrombosis (8 of 11* vs *2 of 16; *p* = 0.03).^10^ In a prospective multicenter follow-up study conducted in seven countries in Europe, despite the fact that treatment started early after the appearance of a thrombus, the recanalization rate of PVT was only 37 out of 95 patients (39%). In the same study, the recanalization rate of a splenic vein thrombus and superior mesenteric venous thrombus was 69 (80%) and 76 (73%) of 95 patients, respectively, which was much higher than that of PVT. Besides, at the end of the follow-up term, cavernomas were seen in 38 of 95 patients (40%).^12^ Therefore, these results support the idea that systemic anticoagulation therapy alone might have limited advantages, particularly for cases of extensive PVT. Recently, various studies have reported aspiration thrombectomy, and thrombolysis using mesenteric catheters as treatments for PVT, in addition to systemic anticoagulation therapy.^1–4^

As to the approach to the portal vein, the transjugular,^3^ the trans-splenic,^13^ and the percutaneous transhepatic approach^4^ are known in addition to the transileocolic approach under laparotomy. The percutaneous approach is less invasive than the transileocolic approach; however, the risk of bleeding might be increased. In one report, of 46 patients who underwent the trans-splenic approach, 3 patients (6.5%) had severe bleeding, and 6 patients (13%) had mild bleeding.^13^ In the present case, surgeons recommended a safe, careful, simple, and smooth procedure as the patient had just undergone a major hepatectomy. Therefore, the transileocolic approach was selected. Unlike other approaches, the percutaneous transhepatic approach was in the hepatofugal direction. In cases of thrombotic obstruction of the entire peripheral portal vein, as in the present case, inserting a catheter in the hepatopetal direction is reasonable for removing the PVT.

The biggest advantage of mechanical thrombectomy is that outflow of the portal vein can be obtained. This makes it possible to prevent one of Virchow’s triads, namely, venous stasis, as mentioned above. However, such effects cannot be expected from systemic anticoagulation therapy.

Loss et al reported five PVT patients treated with mechanical thrombectomy, thrombolytic therapy, and anticoagulation therapy. Recanalization was successful in the four patients in which PVT was detected within 6 days after the appearance of clinical symptoms, whereas it was unsuccessful in the one patient in whom PVT was detected after more than 7 days.^1^ Kuboki et al^5^ reported 11 PVT patients who underwent surgical thrombectomy, portal vein stent placement, thrombolytic therapy, and anticoagulation therapy. In seven patients in whom the thrombus was detected by POD 5 (early PVT detection group), the recanalization rate was 100%. In the four cases in which the thrombus was detected after POD 6 (late PVT detection group), the recanalization rate was 50%. In the early PVT detection group, the period until recanalization was 7.1 days, whereas in the late PVT detection group, it was 67.5 days. These studies indicated that the time of treatment was an important factor in the success of PVT treatment. Early detection and treatment not only led to successful PVT removal but also shortened the duration of thrombolysis. Therefore, complications of bleeding due to long-term anticoagulation therapy can be avoided.

In the present case, when abdominal Doppler ultrasound was used to puncture the peripheral dilated bile duct during the PTBD procedure, PVT was detected incidentally (POD 5). This led to reoperation combined with intervention at an early stage, and consequently contributed to complete thrombus disappearance and successful recanalization. It is expected that using this treatment method, it would be impossible to achieve good results, such as were obtained in our present case, in cases in which detection is delayed. In our hospital, CECT is routinely performed at POD 4 in patients after hepatectomy. However, PVTs often occur in unexpected cases. In the present case, although bile duct obstruction was suspected, and CT was performed on POD 2, a PVT did not exist at that time. As the PVT was found on POD 5, it was thought to have developed between POD 3 and 5. When PVT is suspected from the patient’s symptoms, physical examination, and laboratory data, an abdominal Doppler ultrasound should be performed for real-time detection of PVT, and CECT should be performed for accurate detection and width measurement of the thrombus. An early treatment strategy can then be planned and interventional treatment can be promptly performed.

## Conclusion

PVT was found in a patient at a relatively early time point after hepatectomy, and blood flow was successfully restored, including that to the intrahepatic portal vein. Complete disappearance of the thrombus was confirmed by CECT 16 days after the reoperation, by thrombolytic and anticoagulation therapy from the mesenteric catheter, in addition to systemic anticoagulation therapy.

## Learning points

1. PVT after hepatectomy is initially asymptomatic and hence its detection is often delayed. However, once acute PVT is detected, accurate evaluation of the progression of PVT should be performed immediately using CECT, and treatments should be started in its early stages.
2. A wide range of PVT types should be considered when performing interventions.
3. The purpose of thrombectomy and aspiration is to recanalize at least the outflow of the portal vein.
